# Screening of Reference Gene for RT-qPCR in *Leymus chinensis* During Environmental Stress Conditions

**DOI:** 10.3390/ijms27146426

**Published:** 2026-07-20

**Authors:** Jinfang Li, Dongli Wan, Jinhua Liu, Chaoqun Zhang, Yongqing Wan

**Affiliations:** 1 Inner Mongolia Key Laboratory of Plants Adversity Adaptation and Genetic Improvement in Cold and Arid Regions, College of Life Sciences, Inner Mongolia Agricultural University, Hohhot 010018, China; 18047150341@163.com (J.L.); liujinhua@czmc.edu.cn (J.L.); 2 Institute of Grassland Research of Chinese Academy of Agricultural Science, Hohhot 010010, China; move118@163.com; 3 Key Lab of Inner Mongolia for Sand Shrubs Fibrosis and Energy Development, Utilization, College of Material Science and Art Design, Inner Mongolia Agricultural University, Hohhot 010018, China; zhangcq@imau.edu.cn

**Keywords:** *Leymus chinensis*, RT-qPCR, reference gene, abiotic stress, hormone treatment, gene expression normalization

## Abstract

Reliable reference genes are critical for ensuring the accuracy of RT-qPCR-based gene expression analysis, especially under environmental stress conditions. In this study, *Leymus chinensis* was used as the experimental material, and eight candidate reference genes—alpha-tubulin (*TUA*), beta-tubulin (*TUB*), glyceraldehyde-3-phosphate dehydrogenase (*GAPDH*), elongation factor 1-alpha (*EF1α*), 18S ribosomal RNA (*18S rRNA*), adenylyl cyclase-associated protein (*CAP*), adenine phosphoribosyl transferase (*APRT*), and actin (*ACT*)—were selected to evaluate their expression stability under cold, drought, heat, NaCl, high pH, wounding, abscisic acid (ABA) and jasmonic acid (JA) treatments. Primer specificity and amplification efficiency were first assessed, and the candidate genes were then comprehensively analyzed using *geNorm*, *NormFinder*, *BestKeeper*, and *RefFinder*. The results showed that the amplification efficiencies of all primers ranged from 95.0% to 107.2%, and the Ct values of the candidate genes ranged from 17.31 to 30.99. Comprehensive analysis using *RefFinder* showed that *ACTIN* was the most stable gene under ABA and NaCl treatments, *EF1α* under heat and wounding treatments, *CAP* under JA and high pH treatments, *APRT* under cold treatment, and *TUB* under drought treatment. *geNorm* analysis indicated that two reference genes were sufficient for accurate normalization under each treatment condition. The reliability of the screening results was further confirmed by expression-level validation of *LcbZIP46*, *LcWRKY5*, and *LcFIN1*. This study provides a stable reference gene system for RT-qPCR-based expression analysis in *Leymus chinensis* under environmental stress conditions.

## 1. Introduction

*Leymus chinensis* is a dominant constructive species in northern China’s native grasslands and saline–alkaline meadows, serving dual roles as a high-quality forage with high crude protein content and excellent palatability, as well as a pioneer species for ecological restoration [1,2,3]. The plant spreads vigorously through rhizomes, which helps with soil and water conservation. It also tolerates salt, drought, and poor soil—traits that give it real value in grassland ecological reconstruction, degraded grassland management, and animal husbandry [4]. Relevant studies have shown that *Leymus chinensis* is an important grass species for establishing artificial grasslands, improving forage supply on saline–alkali lands, and supporting the grass–livestock industry [5]. In the context of saline–alkali land use, it is not merely a ‘greening plant’ but rather a candidate species capable of converting low-efficiency saline soils into sustainable forage production systems [6]. Transplanting *L. chinensis* can significantly increase plant density, aboveground biomass, and community structural complexity in saline-alkalized grasslands, thus conferring clear ecological restoration value [5,7,8]. However, the utilization of *Leymus chinensis* for grassland restoration and livestock production is severely constrained by its poor reproductive performance, which constitutes the core bottleneck for its reliable establishment, propagation, and high-yield maintenance under actual saline–alkali soil conditions [3].

Saline–alkali stress, especially the combined effects of salt, alkaline pH, ion toxicity, and osmotic stress, significantly inhibits seed germination, seedling root elongation, photosynthesis, and biomass accumulation of *L. chinensis*. Under severe saline–alkali conditions, direct seeding and seedling establishment are particularly vulnerable [8]. Mixed salt–alkali stress significantly reduces the germination rate, germination potential, and vigor index of *Leymus chinensis* seeds, while simultaneously inhibiting seedling growth and increasing cell membrane permeability [9]. Environmental stresses, such as drought, heat, salinity, cold and wounding, are important factors limiting the growth and productivity of grassland plants. These stresses can cause damage to plant membrane systems, protein denaturation, and the accumulation of reactive oxygen species (ROS), thereby inhibiting plant growth and resulting in yield losses [10]. Long-term exposure to natural drought and overgrazing has impeded vegetation recovery in the Inner Mongolian steppe, inducing progressive plant dwarfism and, in some regions, desertification. When subjected to stresses such as salinity, drought, and overgrazing, the endogenous abscisic acid (ABA) content in *Leymus chinensis* increases significantly [11]. In response, exogenous application of ABA can effectively alleviate the inhibitory effect of salt–alkali stress on the growth of *L. chinensis*. Jasmonic acid (JA) and its derivatives are core signaling molecules in plants in response to abiotic stresses such as mechanical wounding and insect herbivory [12]. Physical damage caused by mowing and grazing rapidly activates the JA signal transduction pathway in *Leymus chinensis* [13]. Under such complex environmental conditions, where multiple stresses such as salinization, drought, and extreme temperatures occur simultaneously or alternately, the growth, productivity, and restoration capacity of *Leymus chinensis* are greatly inhibited. Consequently, unraveling the molecular basis of its stress adaptation—particularly through gene expression profiling—has become a central focus for both breeding and ecological restoration.

Unraveling the critical gene expression profiles of *Leymus chinensis* involved in stress sensing, osmotic regulation, antioxidant defense, and phytohormone signaling is essential for identifying candidate genes for stress tolerance, deciphering the underlying mechanisms of saline–alkali resistance, and enabling molecular-assisted breeding or targeted gene functional enhancement. Accumulating evidence indicates that gene expression profiling serves as a key bridge between stress-induced phenotypic changes and underlying regulatory networks [14]. In particular, RT-qPCR, with its high sensitivity, specificity, and broad dynamic range, continues to be a cornerstone technique for corroborating transcriptome data, monitoring the expression dynamics of candidate genes, and assessing differential expression across various tissues, time points, and stress conditions [15,16,17]. In RT-qPCR analysis, the expression stability of reference genes directly affects the reliability of calculating the relative expression levels of target genes [18]. However, an ideal reference gene that remains stable across different developmental stages, different tissues and organs, different physiological states, and different experimental conditions does not exist [19]. Failure to consider variations in target gene expression, combined with the indiscriminate use of one or a few reference genes, compromises the accuracy of RT-qPCR data and can potentially yield incorrect conclusions [18,20,21]. Therefore, screening stable reference genes for specific species and experimental conditions is a prerequisite for reliable RT-qPCR analysis [22]. Nevertheless, the credibility of RT-qPCR data depends critically on experimental design, RNA purity, primer efficiency, and reference gene stability. Furthermore, the MIQE guidelines mandate comprehensive documentation of all experimental procedures and quality control measures to guarantee that the data are both interpretable and reproducible [23]. Thus, for research into the molecular mechanisms underlying stress tolerance in *Leymus chinensis*, the development of a robust RT-qPCR system that performs reliably under saline, drought, cold, and other stress conditions goes beyond being a routine procedural step—it is the fundamental basis for the eventual success of candidate gene functional validation and stress-tolerance breeding efforts [24].

Although relatively stable reference genes have been identified in *Leymus chinensis* under inorganic phosphorus stress, the resulting normalization system is not directly applicable to other abiotic stresses, including salinity, drought, and cold [25]. In view of the heavy reliance on RT-qPCR for validating miRNAs, target genes, and candidate genes in studies of *Leymus chinensis* responses to salinity and drought, the absence of a systematic assessment of reference genes under these specific stress conditions may undermine the robustness of currently available gene expression data and the derived regulatory networks [26]. This hinders our precise dissection of the key stress-tolerance regulatory network in *Leymus chinensis*.

Based on the above research background, this study selected eight candidate reference genes, namely alpha-tubulin (*TUA*), beta-tubulin (*TUB*), glyceraldehyde-3-phosphate dehydrogenase (*GAPDH*), elongation factor 1-alpha (*EF1α*), 18S ribosomal RNA (*18S rRNA*), adenylyl cyclase-associated protein (*CAP*), adenine phosphoribosyl transferase (*APRT*), and actin (*ACT*). Their expression stability was systematically evaluated under six abiotic stresses and two hormone treatments using four algorithms (*geNorm* [27], *NormFinder* [28], *BestKeeper* [29], and *RefFinder* [30]) to further identify the most stable internal control genes. The present study evaluated reference gene stability under diverse stresses to select the most reliable candidates and their best combinations for RT-qPCR normalization. These findings will facilitate gene functional studies and breeding efforts, and they are expected to enhance the stress tolerance of *L. chinensis* in saline–alkaline environments. Overall, our results underpin future work on stress signaling networks and the generation of improved germplasm.

## 2. Results

### 2.1. Selection of Candidate Reference Genes

Eight candidate reference genes used in the present study were screened from previously reported studies [25]. The candidate genes included alpha-tubulin (*TUA*), beta-tubulin (*TUB*), glyceraldehyde-3-phosphate dehydrogenase (*GAPDH*), elongation factor 1-alpha (*EF1α*), 18S ribosomal RNA (*18S rRNA*), adenylyl cyclase-associated protein (*CAP*), adenine phosphoribosyl transferase (*APRT*), and actin (*ACT*).

### 2.2. Amplification Specificity, Amplification Efficiency, and Expression Profile Analysis of Candidate Reference Genes

The RT-qPCR melting curve results showed that all eight candidate reference genes exhibited a single peak, indicating that each primer pair had good amplification specificity [25]. Primer amplification efficiency is an important indicator for evaluating the reliability of RT-qPCR primers, reflecting the amplification capacity of the target fragment in each PCR cycle. Ideally, when the amplification efficiency is 100%, the product approximately doubles in each cycle [31]. Generally, amplification efficiencies within the range of 90–110% are considered reliable. An excessively low efficiency may be associated with poor primer design, low template quality, or the presence of PCR inhibitors, whereas an excessively high efficiency may indicate problems such as nonspecific amplification, primer–dimer formation, or dilution errors in the standard curve [32,33]. Eight candidate reference genes (*TUA*, *TUB*, *APRT*, *EF1α*, *18S rRNA*, *CAP*, *GAPDH*, and *ACTIN*) and their specific RT-qPCR primer sequences used in this study were retrieved from a previous study on the responses and expression analysis of phosphorus-responsive genes in *Leymus chinensis* under inorganic phosphorus stress [25]. The relevant characteristic parameters, including primer amplification efficiency and amplicon length, were adopted from the data reported in that study. The primer amplification efficiencies of the candidate genes ranged from 95.0% to 107.2%, all of which were within the acceptable range for RT-qPCR analysis.

**Figure 1:**
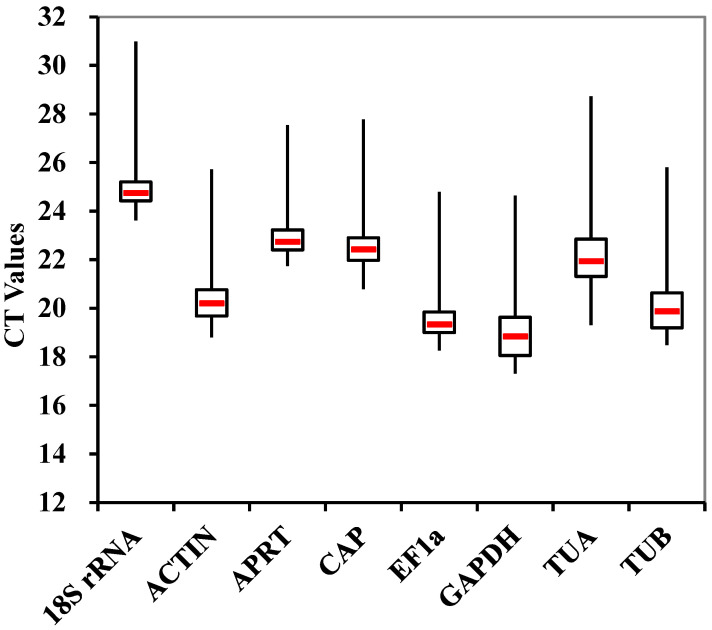
Distribution of cycle threshold (CT) values for eight candidate reference genes. Box-and-whisker plot showing the CT values of eight candidate reference genes across all tested samples. The horizontal red line inside each box indicates the median CT value, the box represents the interquartile range (IQR, 25th to 75th percentiles), and the whiskers extend to the minimum and maximum values. Higher CT values indicate lower transcript abundance.

The expression abundance of the eight candidate reference genes across all samples was analyzed based on Ct values (Figure 1). Ct values are negatively correlated with gene expression abundance; that is, the lower the Ct value, the higher the gene expression level. The results showed that the Ct values of the eight candidate genes ranged from 17.31 to 30.99, with most genes concentrated between 19 and 27. The mean Ct value of *GAPDH* was the lowest (19.68), indicating its relatively high expression abundance, whereas *18S rRNA* had the highest mean Ct value (25.11), suggesting relatively low expression abundance in the samples analyzed in this study. These results indicate that the expression levels of different candidate reference genes vary among *L*. *chinensis* samples subjected to different treatments, and further evaluation using stability algorithms is therefore required.

### 2.3. Expression Stability Analysis of Candidate Reference Genes

To systematically evaluate the expression stability of the eight candidate reference genes under abiotic stress and hormone treatment conditions, four statistical tools, *geNorm*, *NormFinder*, *BestKeeper*, and *RefFinder*, were used for comprehensive analysis in this study.

#### 2.3.1. *geNorm* Stability Analysis

**Figure 2:**
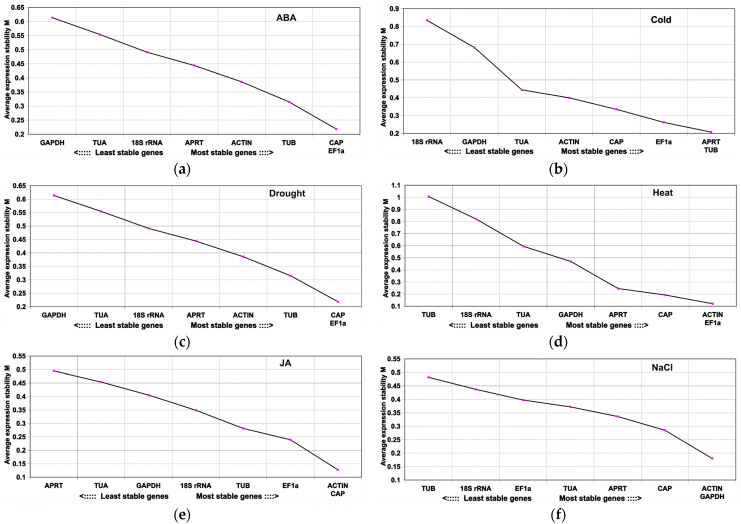

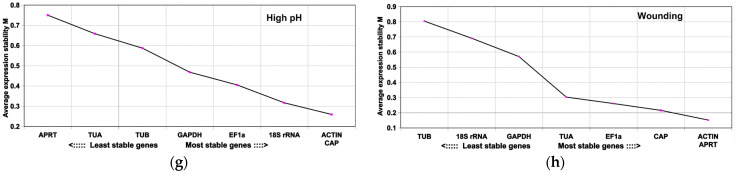
geNorm-based expression stability analysis of candidate reference genes under different abiotic/biotic stress conditions. The average expression stability (M value) of each gene is shown, with lower M values indicating higher expression stability. Genes are ranked from the least stable (left) to the most stable (right) under each condition. (**a**) ABA treatment; (**b**) Cold stress; (**c**) Drought stress; (**d**) Heat stress; (**e**) JA treatment; (**f**) NaCl stress; (**g**) High-pH stress; (**h**) Wounding treatment.

*geNorm* screens stable reference genes by calculating the expression stability value M of candidate reference genes, with a lower M value indicating greater expression stability [34]. The results showed that the M values of all candidate genes were below 1.5, meeting the threshold recommended by *geNorm* [27]. Under ABA and drought treatments, *CAP* and *EF1α* have the highest stability, while *GAPDH* has the lowest stability. Under cold stress, *APRT* and *TUB* are the most stable genes, whereas *18S rRNA* is the least stable. *ACTIN* shows relatively high stability under heat, JA, NaCl, high pH and wounding treatments. Conversely, *TUB* and *GAPDH* show large variations in stability under different treatment conditions (Figure 2). In the present study, the M values of the eight candidate genes were all below 1.5, demonstrating that each was suitable as a reference gene.

**Figure 3:**
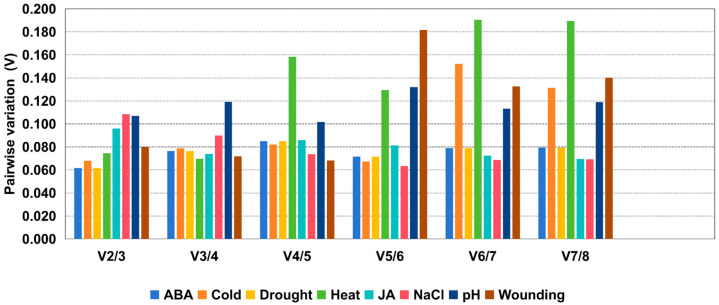
*geNorm* pairwise variation (Vn/n+1) analysis for optimal reference gene number under stress conditions (V < 0.15 threshold). Treatments are color-coded.

*geNorm* can also compute the pairwise variation V of the normalization factors after introducing an extra reference gene, and the optimal number of reference genes can be determined based on Vn/Vn+1 [27]. The pairwise variation (Vn/Vn+1) was used to decide the optimal number of reference genes, with 0.15 as the criterion for deciding if additional reference genes are needed. The results show that under ABA, cold, heat, JA, NaCl, high pH and wounding treatments, the V2/3 values are all less than 0.15, indicating that two reference genes are sufficient for expression normalization. For all samples, the V2/3 values are less than 0.15, suggesting that two reference genes are the most suitable for normalization. However, under drought stress, the V4/5 value exceeds 0.15, implying that more reference genes are required for combined normalization (Figure 3). This suggests that gene expression regulation under drought stress is more complicated and needs a larger number of reference genes.

#### 2.3.2. *NormFinder* Stability Analysis

*NormFinder* can not only evaluate the expression variation of candidate reference genes but also simultaneously consider intra- and inter-group variations, thereby identifying the most stably expressed reference genes [35]. The *NormFinder* analysis results differed slightly from those obtained using *geNorm*. Under ABA treatment, the top three most stable genes were *ACT*, *CAP*, and *EF1α*; under cold treatment, they were *APRT*, *TUB*, and *EF1α*; under drought treatment, they were *TUB*, *ACT*, and *EF1α*; under heat treatment, they were *GAPDH*, *TUA*, and *EF1α*; under JA treatment, they were *EF1α*, *CAP*, and *TUB*; under NaCl treatment, they were *ACT*, *CAP*, and *GAPDH*; under high pH treatment, they were *CAP*, *ACT*, and *EF1α*; and under wounding treatment, they were *CAP*, *EF1α*, and *TUA* (Table 1). Overall, although the stability rankings obtained using *NormFinder* and *geNorm* were not completely consistent, they showed a high degree of agreement in identifying unstable genes under different treatment conditions.

#### 2.3.3. *BestKeeper* Stability Analysis

**Figure 4:**
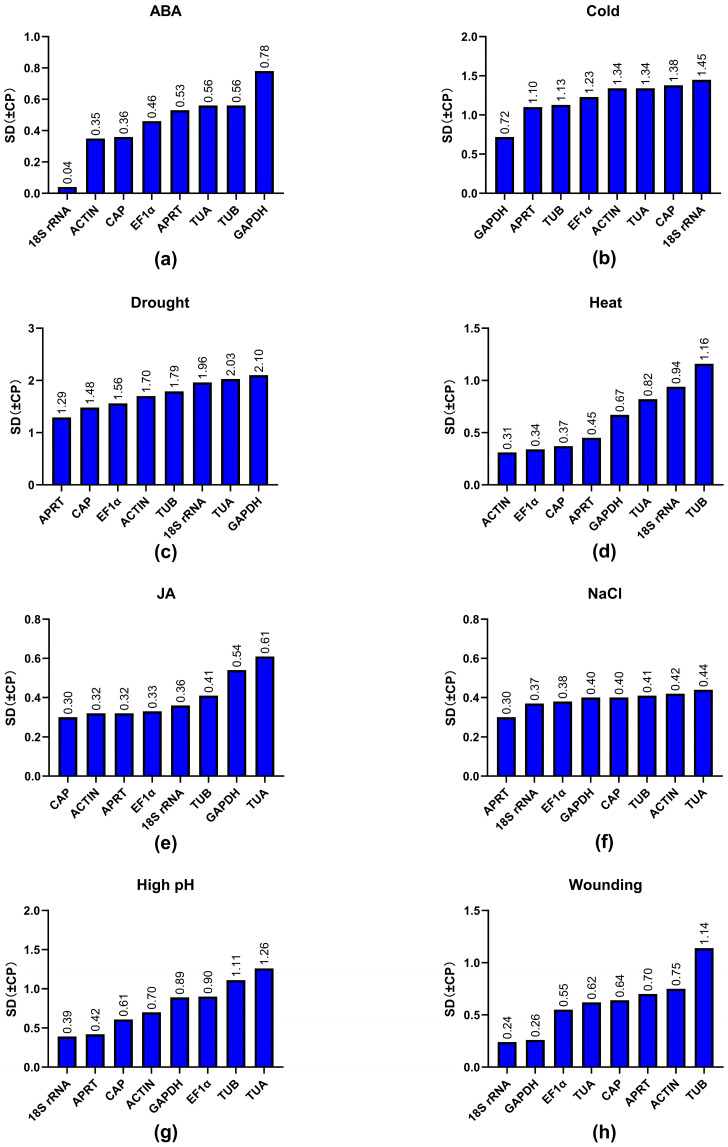
*BestKeeper*-based stability ranking of candidate reference genes under different treatments. Genes are sorted by the standard deviation (SD, ±CP) of their Ct values, with lower SD corresponding to higher expression stability. Treatments include (**a**) ABA, (**b**) Cold, (**c**) Drought, (**d**) Heat, (**e**) JA, (**f**) NaCl, (**g**) High pH, and (**h**) Wounding.

*BestKeeper* mainly evaluates expression stability based on the standard deviation (SD) and coefficient of variation (CV) of the Ct values of candidate genes, with lower SD and CV values indicating more stable gene expression [36]. *BestKeeper* analysis showed that *18S rRNA* had the lowest SD values and relatively high stability under ABA, high pH, and wounding treatments. *GAPDH* showed relatively high stability under cold treatment, whereas *APRT* ranked first under drought treatment; however, its SD value was greater than 1, suggesting substantial expression variation in drought-treated samples and indicating that it is not suitable as a stable reference gene when used alone. *ACT* showed relatively high stability under heat treatment, *CAP* under JA treatment, and *APRT* under NaCl treatment (Figure 4). In terms of identifying the least stable genes, the *BestKeeper* results were generally consistent with those of *NormFinder*, except under JA, NaCl, and high pH treatments.

#### 2.3.4. Comprehensive Stability Analysis Using *RefFinder*

*RefFinder* can integrate the results obtained using the Δ*Ct* method, *NormFinder*, *BestKeeper*, and *geNorm* to comprehensively rank candidate reference genes [37]. *RefFinder* analysis showed that *ACT* was the most stably expressed gene under ABA and NaCl treatments, whereas the least stable genes were *GAPDH* and *TUB*, respectively. Under heat and wounding treatments, *EF1α* was the most stably expressed gene, while *TUB* was the least stable gene in both treatments. Under JA and high pH treatments, *CAP* was the most stably expressed gene, whereas *TUA* was the least stable gene in both treatments. Under cold treatment, *APRT* was the most stably expressed gene, while *18S rRNA* was the least stable. Under drought treatment, *TUB* was the most stably expressed gene, whereas *GAPDH* was the least stable (Table 2). The comprehensive results further indicate that the stability of candidate reference genes in *Leymus chinensis* varies markedly under different treatment conditions, and suitable reference genes should therefore be selected according to specific experimental conditions.

### 2.4. Verification of the Selected Reference Genes

To validate the reliability of the selected stable reference genes, the target genes *LcbZIP46* [38], *LcWRKY5* [39], and *LcFIN1* [40] were used as candidate genes. Specifically, a reference gene with poor stability, a single reference gene with relatively good stability, and the optimal combination of reference genes were respectively employed to normalize the expression levels of *LcbZIP46*, *LcWRKY5*, and *LcFIN1* under different treatment conditions.

**Figure 5:**
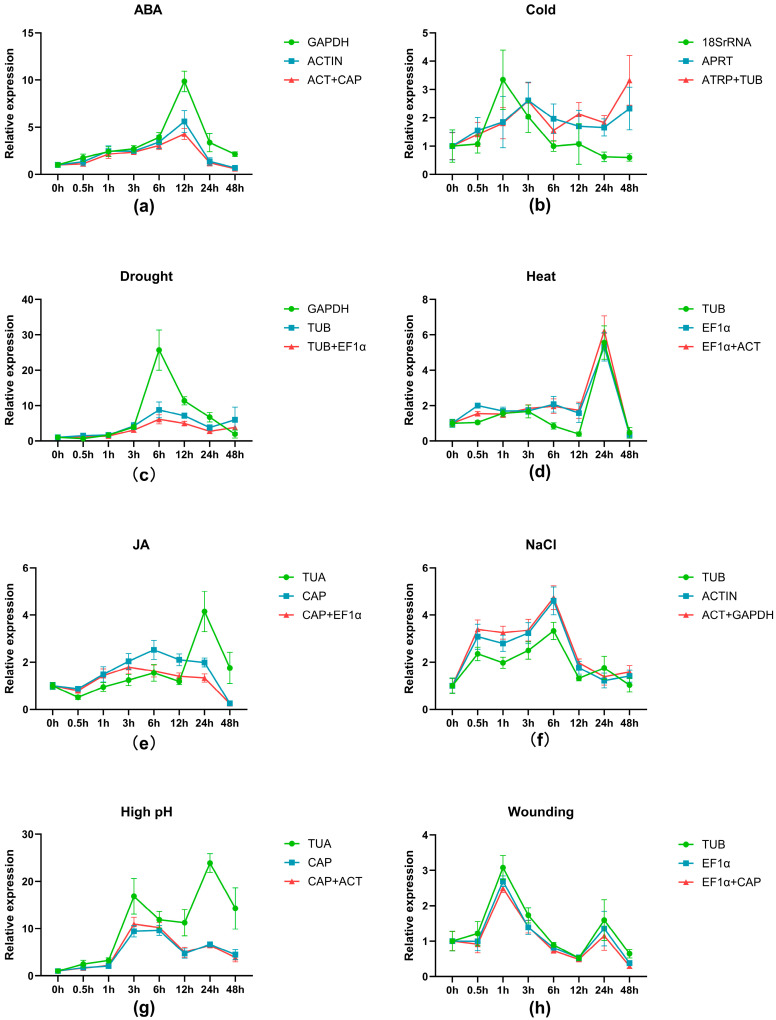
Relative expression levels of *LcbZIP46* under various stress treatments, normalized using different reference gene normalization strategies. Three normalization approaches are shown: the most stable single gene (green), the second most stable single gene (blue), and the optimal reference gene pair (red). Treatments include (**a**) ABA, (**b**) Cold, (**c**) Drought, (**d**) Heat, (**e**) JA, (**f**) NaCl, (**g**) High-pH, and (**h**) Wounding.

Expression analysis of *LcbZIP46* under different conditions revealed that its transcript levels were upregulated following ABA, cold, drought, heat, JA, NaCl, and high-pH treatments, whereas they decreased after wounding treatment (Figure 5). When the optimal reference gene combination was used for normalization, *LcbZIP46* exhibited similar expression trends. In contrast, normalization with the least stable reference gene led to divergent expression patterns. For example, under cold treatment (Figure 5b), using *APRT* or *APRT*+*TUB* as reference genes consistently showed upregulation of *LcbZIP46*, while using *18S rRNA* resulted in downregulation. Moreover, at 12 h after ABA treatment (Figure 5a), normalization with *ACT* and *ACT*+*CAP* increased *LcbZIP46* expression by approximately 5-fold, whereas normalization with *GAPDH* increased it by about 10-fold.

**Figure 6:**
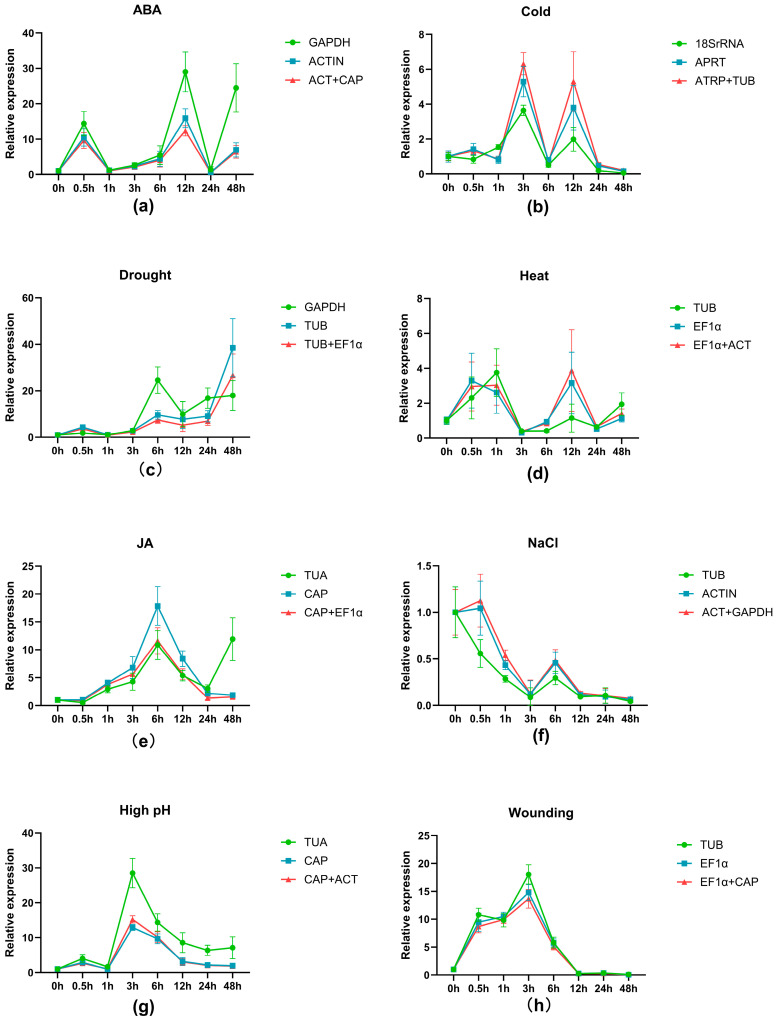
Relative expression levels of *LcWRKY5* under various stress treatments, normalized using different reference gene normalization strategies. Three normalization approaches are shown: the most stable single gene (green), the second most stable single gene (blue), and the optimal reference gene pair (red). Treatments include (**a**) ABA, (**b**) Cold, (**c**) Drought, (**d**) Heat, (**e**) JA, (**f**) NaCl, (**g**) High pH, and (**h**) Wounding.

Expression analysis of *LcWRKY5* revealed that its transcript levels were upregulated under ABA, drought, heat, JA, high pH, and wounding treatments, whereas they were significantly downregulated under NaCl and cold treatments (Figure 6). However, the expression patterns obtained with the most stable reference gene and the optimal combination also differed from those obtained with the least stable reference gene. For example, after 3 h of high-pH treatment (Figure 6g), *LcWRKY5* expression was upregulated approximately 10-fold when normalized with the most stable reference gene or the optimal combination, whereas it was upregulated approximately 30-fold with the least stable reference gene.

**Figure 7:**
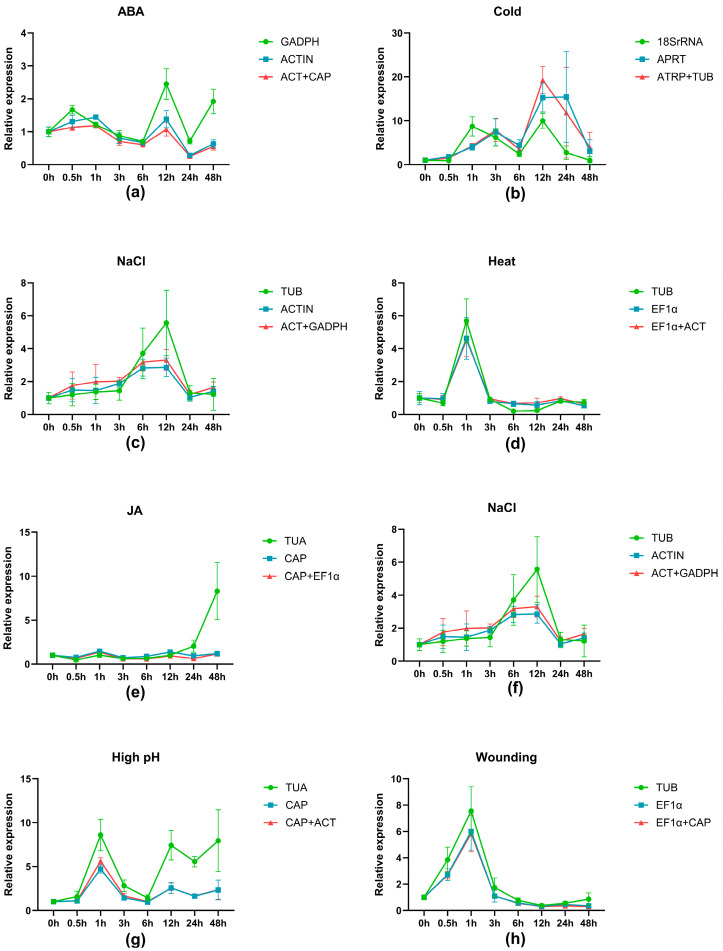
Relative expression levels of *LcFIN1* under various stress treatments, normalized using different reference gene normalization strategies. Three normalization approaches are shown: the most stable single gene (green), the second most stable single gene (blue), and the optimal reference gene pair (red). Treatments include (**a**) ABA, (**b**) Cold, (**c**) Drought, (**d**) Heat, (**e**) JA, (**f**) NaCl, (**g**) High pH, and (**h**) Wounding.

Expression analysis of *LcFIN1* revealed that its transcript levels were upregulated following cold, drought, NaCl, and high pH treatments, whereas they were downregulated under ABA, heat, and wounding treatments (Figure 7). Overall, only minor differences were observed when using the most stable reference gene and the optimal combination, whereas the use of the least stable reference gene resulted in distinct expression patterns. These findings indicate that the selection of stable reference genes for RT-qPCR in *Leymus chinensis* is reliable across different experimental conditions.

## 3. Discussion

At present, RT-qPCR is widely used for the quantification and detection of plant gene expression. For reference genes used to normalize gene expression data, their expression stability directly affects the accuracy of RT-qPCR analysis. Traditionally, housekeeping genes such as *ACTIN*, *GAPDH*, *TUB*, *EF1α*, and *18S rRNA* have often been used for expression normalization; however, increasing evidence has shown that these genes do not maintain stable expression across all species, tissues, or stress conditions [41]. Therefore, systematic screening and validation of candidate reference genes before RT-qPCR analysis under specific species and treatment conditions is a necessary step to ensure the reliability of the results. In recent years, an increasing number of studies on reference gene validation have been reported in plants such as rice [42,43,44], peanut [45], soybean [46], petunia hybrids [47], citrus [48], and poplar [49]. In perennial forages, graminaceous crops, and various economically important plants, screening reference genes using algorithms such as *geNorm*, *NormFinder*, *BestKeeper*, and *RefFinder* has become a commonly used technical approach [50,51,52]. In this study, *L. chinensis* was used as the experimental material, and the expression stability of eight candidate reference genes was systematically evaluated under eight treatment conditions, including ABA, cold, drought, heat, JA, NaCl, high pH, and wounding. These results provide a reference for gene expression analysis related to stress responses in *L. chinensis*.

In this study, the amplification efficiencies of the eight candidate reference genes ranged from 95.0% to 107.2%, all of which were within the acceptable range for RT-qPCR analysis [25]. In addition, all melting curves showed a single peak, indicating that the designed primers had good amplification specificity [25]. The Ct values of the candidate genes ranged from 17.31 to 30.99, indicating that these genes could be effectively detected in *L. chinensis* samples [25]. Among them, *GAPDH* had a relatively low mean Ct value, suggesting high expression abundance, whereas *18S rRNA* had a relatively high mean Ct value, indicating relatively low expression abundance. It should be noted that Ct values only reflect gene expression abundance and do not directly represent expression stability; expression stability still needs to be comprehensively evaluated based on expression fluctuations across different treatments and time points. Similar studies have also indicated that the Ct/Cq values of candidate reference genes can be used for the preliminary assessment of expression abundance, but final reference gene selection still relies on multi-algorithm stability analysis [53,54]. The evaluation results of reference gene stability differed to some extent among different algorithms, which was a relatively obvious phenomenon in this study. *geNorm* mainly calculates the M value based on pairwise variation among candidate genes and further determines the required number of reference genes using Vn/Vn+1. *NormFinder* simultaneously considers intra- and inter-group variations, whereas *BestKeeper* ranks genes mainly based on the standard deviation and coefficient of variation in Ct values. *RefFinder* can integrate the results of multiple algorithms to generate a comprehensive ranking [55,56,57]. Because these algorithms are based on different calculation principles, discrepancies in the final rankings are normal [58].

In many plant species, optimal reference genes have been identified under various experimental conditions. *ACT* is the most stable reference gene in birch under salt stress, whereas *TUB* is the most stable reference gene under osmotic stress [59]. In spinach, *18S rRNA* was identified as the optimal reference gene in roots, stems, leaves, flowers, and seedlings, as well as in leaves and roots under salt stress [60]. In *A. bidentata* under hormone, NaCl, and drought treatments, *TUB* was also identified as an unstable reference gene compared with other candidate reference genes, such as *18S rRNA*, *EF1α*, *GAPDH*, *TUBβ*, *UBC*, and *UBQ* [61]. *ACT* was the most stable reference gene in *Salsola collina* under heat treatment [62]. However, under many experimental conditions, *ACTIN*, *18S rRNA*, and *GAPDH* are not suitable reference genes for normalization in papaya [63]. Previous studies have screened reference genes in different tissues of *L*. *chinensis*, but reference gene screening in *L. chinensis* under abiotic stress has not yet been reported. Therefore, it is essential to select stably expressed genes in *L. chinensis* under abiotic stress as internal references. In this study, the stability rankings obtained using *NormFinder*, *BestKeeper*, and *geNorm* differed under some treatments. However, genes with relatively high stability were generally concentrated among *ACTIN*, *CAP*, *EF1α*, *APRT*, and *TUB*, suggesting that these genes have potential application value under specific stress treatments in *L*. *chinensis*. The results showed that the stability of candidate reference genes in *L*. *chinensis* was clearly stress-specific, and no universal reference gene suitable for all treatment conditions was identified. According to the comprehensive ranking by *RefFinder*, *ACTIN* was the most stably expressed gene under ABA and NaCl treatments; *EF1α* was the most stably expressed gene under heat and wounding treatments; *CAP* was the most stably expressed gene under JA and high-pH treatments; *APRT* was the most stably expressed gene under cold treatment; and *TUB* was the most stably expressed gene under drought treatment. These results indicate that the same reference gene may exhibit different levels of stability under different stress conditions, and the use of a single reference gene across all treatments may lead to biased analysis of target gene expression levels. In studies of *Rumex patientia* under multiple abiotic stresses, the recommended reference gene combinations also differed among different stress conditions [64]. Therefore, screening stable reference genes separately for different stress conditions is an important principle that should be followed in gene expression studies of stress responses in *L*. *chinensis*.

Compared with the use of a single reference gene, the combined use of multiple reference genes can generally reduce the influence of expression fluctuations of individual genes on normalization results, thereby improving the accuracy and stability of RT-qPCR data. In this study, *geNorm* pairwise variation analysis showed that the V2/V3 values under the treatment conditions were below 0.15, indicating that the use of two reference genes was sufficient to meet the requirements for RT-qPCR data normalization in *L. chinensis* under environmental stress conditions. According to the *geNorm* recommendations, *CAP/EF1α* is recommended under ABA and drought treatments; *APRT/TUB* under cold treatment; *ACTIN/EF1α* under heat treatment; *ACTIN/CAP* under JA and high-pH treatments; *ACTIN/GAPDH* under NaCl treatment; and *ACTIN/APRT* under mechanical wounding treatment. This result is consistent with the conclusions of most recent studies, which suggest that dual or multiple reference gene combinations should be preferentially used for normalization analysis when conditions permit.

To validate the reliability of the selected reference genes, we quantified the expression of three known stress-responsive transcription factors in *L. chinensis*—*LcbZIP46*, *LcWRKY5*, and *LcFIN1*—under the respective stress conditions. bZIP transcription factors are involved in plant growth and development, induce plant hormone responses, and regulate defense mechanisms under abiotic stresses such as drought, high salinity, and temperature stress, as well as pathogen invasion [65]. *LcFIN1* responds to low temperature, and plants overexpressing *LcFIN1* exhibit significantly superior multiple cold-tolerance phenotypes compared with wild-type plants under cold stress [40]. WRKY transcription factors regulate the transcriptional mechanisms, signaling pathways, and hormonal crosstalk networks involved in plant disease resistance defense [66]. Using the reference genes identified in this study, we accurately characterized the expression profiles of *LcbZIP46*, *LcWRKY5*, and *LcFIN1* under salt–alkali, drought, and cold stress, respectively. *LcbZIP46* showed significant induction under saline–alkali stress, consistent with its established function in alkali tolerance [38]. *LcWRKY5* was markedly upregulated by drought, corroborating previous reports that its overexpression enhances drought tolerance in transgenic Arabidopsis via upregulation of *DREB2A* and *RD29A* through ABA-independent pathways [39]. Previous studies have shown that *LcFIN1* is strongly induced by cold stress [40]. In the present study, using the validated reference genes, we reliably detected the induced expression of *LcFIN1* under cold treatment. This cross-validation not only supports the suitability of our selected reference genes for cold-stress studies, but also provides a foundation for future functional dissection of the *LcFIN1* regulatory module and its application in breeding cold-tolerant *L. chinensis* germplasm. The observed induction of *LcbZIP46*, *LcWRKY5*, and *LcFIN1* under their respective stresses corroborates previous findings and confirms that our reference genes provide reliable normalization for stress-response studies. More importantly, this cross-validation supports the utility of our normalization system for future functional genomics studies aimed at dissecting the regulatory networks governing stress tolerance in *L. chinensis*. Taken together, our results reveal that *LcbZIP46*, *LcWRKY5* and *LcFIN1* respond to multiple biotic and abiotic stress stimuli, highlighting their potential as key functional genes mediating stress resistance in *Leymus chinensis*. The stable reference genes chosen in this study will enable reliable normalization and quantification of gene expression under multiple stresses in future studies.

## 4. Materials and Methods

### 4.1. Plant Material Collection

The *L. chinensis* seedlings used in this experiment were grown from seeds of the cultivar Jisheng No. 4. The seeds were sown in nutrient pots containing a mixture of vermiculite and nutrient soil at a ratio of 2:1 and germinated at 22 °C under plastic film coverage. The pots used for plant cultivation had dimensions of approximately 7.5 cm × 7.5 cm × 7.5–8 cm (length × width × height). A total of 36 seeds were sown in each pot. After germination, seedling roots were gently rinsed, and the seedlings were transferred to Hoagland’s nutrient solution for cultivation. The culture conditions were set at 22 °C with a photoperiod of 16 h light/8 h dark. After 30 days of cultivation, the seedlings were subjected to subsequent stress treatments. For each treatment, 120 plants were used, and the experiment was performed in triplicate.

### 4.2. Stress Treatments

In this study, *L. chinensis* seedlings were subjected to cold, wounding, high-pH, NaCl, JA, ABA, drought, and heat treatments. Among these treatments, wounding, cold, and heat stresses were applied directly to plants grown in pots. For the other five treatments (ABA, JA, NaCl, high-pH, and drought), the seedlings were carefully washed after germination and transferred to Hoagland nutrient solution, where they were cultured for 30 days before stress imposition. All different treatment conditions were implemented mainly following previously reported methods [67]. For the mechanical wounding treatment, the middle-lower part of the leaves was wounded using a sterile needle, and the plants were then placed in a growth chamber. For cold and heat treatments, the plants were incubated at 4 °C and 42 °C, respectively. For drought treatment, the plants were rinsed with distilled water, blotted dry, placed on filter paper, and incubated in a growth chamber. For ABA, JA, high-pH, and salt stress treatments, the plants were immersed in solutions containing 100 μM ABA, 100 μM JA, pH 10 (adjusted with 200 mM NaHCO_3_ buffer), and 300 mM NaCl, respectively. For each treatment, samples were collected at 0, 0.5, 1, 3, 6, 12, 24, and 48 h, with the 0 h samples used as controls. Whole plants were collected for RNA extraction, and three independent biological replicates were performed for each treatment. The collected samples were immediately frozen in liquid nitrogen and then stored at −80 °C for subsequent RNA extraction.

### 4.3. Total RNA Extraction and cDNA Synthesis

Using the MiniBEST Plant RNA Extraction Kit (TaKaRa, Kyoto, Japan), total RNA from *L. chinensis* was extracted and its concentration and purity were evaluated through A_260_/A_280_ ratio and agarose gel electrophoresis. Qualified RNA samples were required to have an A_260_/A_280_ ratio ranging from 1.8 to 2.1 and an A_260_/A_230_ ratio ≥ 1.8. Subsequently, cDNA was synthesized using the PrimeScript™ RT reagent Kit with gDNA Eraser (TaKaRa) after 16-fold dilution, which served as a template for synthesis.

RT-qPCR was carried out using the SYBR Green fluorescent dye method. The RT-qPCR thermal program was set as follows: initial denaturation at 94 °C for 30 s, followed by 40 cycles of 94 °C for 5 s, 60 °C for 15 s and 72 °C for 10 s (fluorescence signal collected at extension step). After cycling, samples were held at 40 °C for 10 s, then subjected to melting curve analysis for 15 s to verify specific amplification. Each cDNA sample was run in triplicate as technical replicates. The relative expression levels of genes were calculated using the 2^−ΔΔCt^ method.

### 4.4. Selection of Reference Genes and Primer Design

The eight candidate reference genes used in this study were all derived from previous literature, namely *LcTUA*, *LcTUB*, *LcAPRT*, *LcEF1α*, *Lc18S rRNA*, *LcCAP*, *LcGAPDH*, and *LcACT* [25]. Primer Premier 5.0 software was used to analyze the sequences of these eight genes and to design primers for RT-qPCR [25].

## 5. Conclusions

In summary, this study systematically evaluated the expression stability of eight candidate reference genes in *L. chinensis* under six abiotic stress conditions and two hormone treatments, identifying suitable single reference genes and optimal two-gene normalization pairs for each specific treatment condition. The results demonstrated that reference-gene stability in *L. Chinensis* is highly condition-dependent. Selecting appropriate stable reference genes for different stress conditions—particularly prioritizing two-gene combinations for normalization—is therefore critical for improving the accuracy of RT-qPCR quantification. Based on *geNorm* analysis, the recommended two-gene normalization pairs were *CAP*/*EF1α* for ABA and drought treatments, *APRT*/*TUB* for cold stress, *ACTIN*/*EF1α* for heat stress, *ACTIN*/*CAP* for JA and high-pH treatments, *ACTIN*/*GAPDH* for NaCl stress, and *ACTIN*/*APRT* for wounding. According to the comprehensive ranking from *RefFinder*, the recommended single reference genes were *ACTIN* for ABA and NaCl treatments, *APRT* for cold stress, *TUB* for drought stress, *EF1α* for heat stress and wounding, and *CAP* for JA and high-pH treatments. Overall, the selection of treatment-specific stable reference genes—especially the preferential use of two-gene normalization pairs—is essential for ensuring accurate RT-qPCR quantification in *L. chinensis*. Collectively, this study provides a reliable reference-gene selection framework for gene expression analysis in *L. chinensis* exposed to abiotic stresses and phytohormone treatments.

## Figures and Tables

**Table 1:** NormFinder-based stability ranking of candidate reference genes under different stress conditions. Genes are ranked from most (Rank 1) to least (Rank 8) stable, with lower stability values indicating higher expression stability.

Rank	ABA	Cold	Drought	Heat	JA	NaCl	High pH	Wounding
1	ACTIN	APRT	TUB	GAPDH	EF1a	ACTIN	CAP	CAP
Stability value	0.086	0.072	0.113	0.285	0.084	0.149	0.058	0.073
2	CAP	TUB	ACTIN	TUA	CAP	CAP	ACTIN	EF1α
Stability value	0.094	0.109	0.180	0.353	0.124	0.164	0.110	0.074
3	EF1α	EF1α	EF1α	EF1α	TUB	GAPDH	EF1α	TUA
Stability value	0.223	0.164	0.200	0.372	0.133	0.184	0.113	0.112
4	TUB	CAP	CAP	APRT	ACTIN	APRT	18S rRNA	APRT
Stability value	0.353	0.297	0.273	0.393	0.144	0.185	0.327	0.119
5	18S rRNA	TUA	18S rRNA	ACTIN	18S rRNA	TUA	GAPDH	ACTIN
Stability value	0.360	0.402	0.295	0.443	0.287	0.199	0.426	0.249
6	APRT	ACTIN	TUA	CAP	GAPDH	EF1a	TUB	GAPDH
Stability value	0.389	0.470	0.381	0.489	0.350	0.267	0.457	0.718
7	TUA	GAPDH	APRT	18S rRNA	TUA	18S rRNA	TUA	18S rRNA
Stability value	0.454	0.761	0.455	0.734	0.367	0.338	0.582	0.720
8	GAPDH	18S rRNA	GAPDH	TUB	APRT	TUB	APRT	TUB
Stability value	0.637	0.820	0.492	1.036	0.375	0.375	0.646	0.762

**Table 2:** Comprehensive ranking of reference genes based on *RefFinder* analysis.

Rank	ABA	Cold	Drought	Heat	JA	NaCl	High pH	Wounding
1	ACTIN	APRT	TUB	EF1a	CAP	ACTIN	CAP	EF1a
Geomean of ranking values	1.19	1.41	1.97	1.57	1.41	1.63	1.32	2.21
2	CAP	TUB	EF1a	ACTIN	EF1a	GAPDH	ACTIN	CAP
Geomean of ranking values	1.41	2.06	2.06	1.78	1.86	2.45	2.00	2.34
3	EF1a	EF1a	CAP	GAPDH	ACTIN	CAP	18S rRNA	APRT
Geomean of ranking values	3.22	2.45	2.38	3.34	2.45	2.78	2.63	2.91
4	18S rRNA	GAPDH	ACTIN	APRT	TUB	APRT	EF1a	ACTIN
Geomean of ranking values	4.40	4.30	3.13	3.72	4.12	2.83	3.83	3.64
5	APRT	CAP	APRT	CAP	18S rRNA	EF1a	GAPDH	TUA
Geomean of ranking values	4.68	4.60	3.96	3.83	5.00	5.05	5.00	3.94
6	TUB	ACTIN	18S rRNA	TUA	APRT	18S rRNA	APRT	18S rRNA
Geomean of ranking values	5.63	5.48	5.48	4.56	5.66	5.12	5.66	4.30
7	TUA	TUA	TUA	18S rRNA	GAPDH	TUA	TUB	GAPDH
Geomean of ranking values	6.74	5.48	6.48	7.00	6.24	5.62	6.24	4.56
8	GAPDH	18S rRNA	GAPDH	TUB	TUA	TUB	TUA	TUB
Geomean of ranking values	8.00	8.00	8.00	8.00	7.24	7.44	7.24	8.00

## Data Availability

The original contributions presented in this study are included in the article. Further inquiries can be directed to the corresponding author.
